# Automated mitochondrial oxygen consumption (mitoVO_2_) analysis via a bi-directional long short-term memory neural network

**DOI:** 10.1007/s10877-025-01291-1

**Published:** 2025-03-30

**Authors:** C. J. de Wijs, J. R. Behr, L. W. J. M. Streng, M. E. van der Graaf, F. A. Harms, E. G. Mik

**Affiliations:** 1 Department of Anesthesiology, Erasmus Medical Center, Rotterdam, the Netherlands; 2 Faculty of Mechanical Engineering, Delft University of Technology, Delft, the Netherlands

**Keywords:** Mitochondrial oxygenation, Mitochondrial oxygen consumption, Automated analyses, Bi-directional long short-term memory neural network

## Abstract

Monitoring in vivo mitochondrial oxygen tension (mitoPO_2_) enables the measurement of mitochondrial oxygen consumption (mitoVO_2_), providing deeper insights into the skin’s mitochondrial environment. However, current mitoVO_2_ analysis often relies on manual identification of start and end points, which introduces substantial inter-user variability. Addressing this limitation is crucial for broader adoption, comparability, and reproducibility across research groups. Therefore, the aim of this study was to develop a neural network–based software that automatically analyzes mitoVO_2_. A Bi-directional Long Short-Term Memory neural network was trained on 125 mitoPO_2_ measurement sequences and optimized through Bayesian optimization. It identifies start points and measurement periods, then applies a modified Michaelis-Menten fit to calculate mitoVO_2_. This framework, embedded in automated software, was validated against the consensus of 3 raters. Bayesian optimization yielded an overall network performance of 94.2% on the test set. The neural network identified 91% of mitoVO_2_ start points within a ± 5-sample range of the manual consensus. Mean mitoVO_2_ values for the consensus and software were 6.56 and 6.63 mmHg s^− 1^, respectively, corresponding to a bias of -0.057 mmHg s^− 1^. Multiple runs of the network on the same dataset produced identical results, confirming consistency and eliminating inter-user variability. The developed neural network–based software automatically and consistently analyzes mitoVO_2_ measurements, substantially reducing reliance on subjective judgments. By enabling a standardized approach to mitoVO_2_ analysis, this tool improves data comparability and reproducibility across research settings. Future work will focus on further refining precision and extending functionality through multi-center collaborations.

**Supplementary Information:**

The online version contains supplementary material available at 10.1007/s10877-025-01291-1.

## Introduction

In recent years, the pathophysiology of mitochondria and the potential impact of mitochondrial dysfunction on various diseases have attracted growing scientific interest [1–4]. This has resulted in mitochondria increasingly becoming a target in treatment strategies for various illnesses [5, 6]. However, there are few methods to examine mitochondrial function. Moreover, existing techniques such as high-resolution respirometry are ex vivo, resulting in a measurement environment that is different from the mitochondria’s natural setting [7, 8]. Whilst magnetic resonance spectroscopy (MRS) is capable of measuring in vivo mitochondrial function it is expensive and time consuming [9]. This underscores the necessity of in vivo mitochondrial function measurements which are not as costly and time consuming as MRS and which are able to evaluate the in situ state of mitochondria.

The Cellular Oxygen METabolism (COMET^®^) monitor, a CE-marked measuring device developed by Photonics Healthcare (Utrecht, NL), facilitates in vivo measurement of mitochondrial oxygen tension (mitoPO_2_) in the skin. The measurements are based on the protoporphyrin IX-triplet state lifetime technique (PpIX-TSLT) [10]. The principle behind this technique revolves around the oxygen-dependent optical properties of protoporphyrin IX (PpIX) [10–12]. To facilitate this technique, endogenous PpIX levels are increased by administering the precursor 5-aminolevulinic acid (ALA).

**Fig. 1:**
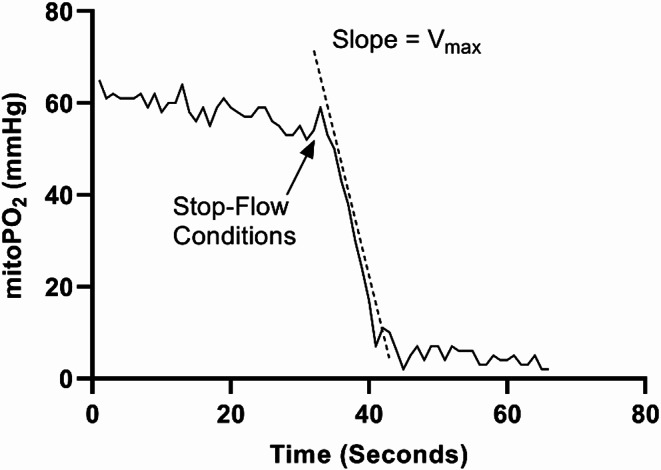
Time course of mitoPO_2_ during mitoVO_2_ measurement and illustration of slope indicating V_max_ of mitoVO_2_. Abbreviations: mitoPO_2_; mitochondrial oxygen tension, V_max_; maximal oxygen consumption

**Fig. 2:**
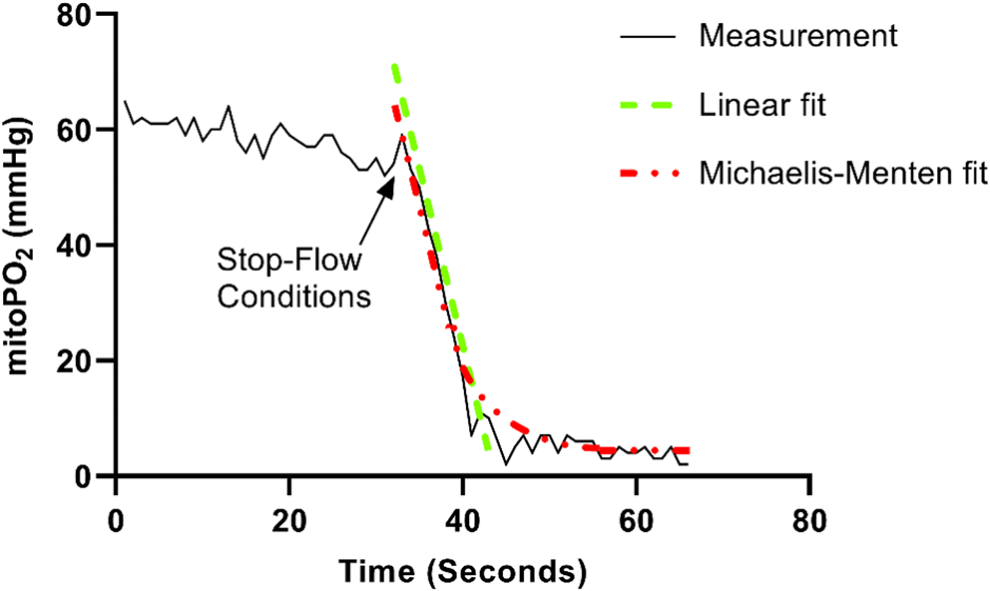
Demonstrative mitoVO_2_ analysis through linear fit compared to Michaelis-Menten fit. Abbreviations: mitoPO_2_; mitochondrial oxygen tension

Since its introduction in 2016, COMET^®^ has been employed in a multitude of clinical studies for measurements of mitoPO_2_ across various clinical settings [13–15]. In addition to mitoPO_2_, COMET^®^ enables the assessment of mitochondrial oxygen consumption (mitoVO_2_), leveraging the principles of oxygen disappearance rate (ODR). The mitoVO_2_ measurements involve inducing a stop-flow condition beneath the measurement probe through localized pressure, leading to a decrease in mitoPO_2_ due to oxygen supply cessation (Fig. 1). The rate at which mitochondrial oxygen is depleted provides insights into oxygen consumption and mitochondrial functionality. Our research group was the first to pioneer this technique for in vivo mitoVO_2_ measurements, initially employing an animal model [16]. Subsequently, the feasibility and application of mitoVO_2_ measurements were expanded to include healthy human volunteers [15] and patients on the intensive care unit [14]. With the introduction of this mitoVO_2_ technique, we developed a method that performs analyses based on an adapted Michaelis-Menten fit procedure. This procedure not only fits the data but also accounts for the diffusion of oxygen into the measurement volume during the stop flow conditions [17]. This innovative approach enables the calculation of maximal oxygen consumption (V_max_), as depicted in Fig. 2.

Baumbach et al. introduced another approach for analyzing mitoVO_2_ through the use of two complementary sigmoid functions [18]. This methodology provides insights into the dynamics of oxygen consumption and subsequent reoxygenation within tissues. Central to this approach is the capability to accurately depict fluctuations mitoPO_2_ over time via specific functional parameters. Another method previously employed, albeit less robust, involves the utilization of a linear fitting procedure.

The diversity of methods for analyzing mitoVO_2_ complicates the comparison of research findings. As pioneers in this field, we strongly advocate for the use of the Michaelis-Menten fit. This method is superior due to its close alignment with core biological processes, including enzyme saturation at increased substrate concentrations. It provides a robust, scientifically grounded, and flexible foundation for precise modeling of mitoVO_2_ in vivo [19].

Alongside these advances, a growing number of studies have demonstrated the power of deep learning for analyzing mitochondrial images and function. For instance, convolutional neural networks have been used to classify mitochondrial organelle movements (fission vs. fusion) in confocal images, providing valuable insights into mitochondrial shape and dynamics [20]. Similarly, deep learning-based approaches have been applied to efficiently categorize mitochondrial images, distinguishing affected (for example, drug-treated) from normal mitochondria [21], as well as to recognize normal vs. diseased cell representations [22]. These studies underscore the potential of advanced computational techniques to enhance the assessment of mitochondrial function and to reduce the subjectivity and manual effort traditionally associated with such analyses.

Unfortunately, our current method for analyzing mitoVO_2_ has its drawbacks. While it is semi-automated, using an in-house developed LabVIEW^®^ program (National Instruments Corp. Austin, Texas, United States) to automatically apply the adapted Michaelis-Menten fit procedure, it requires substantial user intervention. Specifically, users must manually select start and end points for the analysis. This process is not only time-consuming but also introduces a significant limitation due to its dependence on user judgment. These inconsistencies in the selection of start and end points lead to variation in mitoVO_2_ values.

Therefore, we developed a fully automated software for mitoVO_2_ analysis, using a neural network to identify start points of measurements. This approach eliminates the need for user involvement in selecting the start points, thereby reducing inter-user variability. While there is no established gold standard for analyzing mitoVO_2_, our primary objective is to minimize variability between users. This software ensures consistent mitoVO_2_ analysis across various research groups, paving the way for more comparable results across studies. In this study, we discuss the creation and validation of this automated analysis tool.

## Methodology

### Data description

**Fig. 3:**
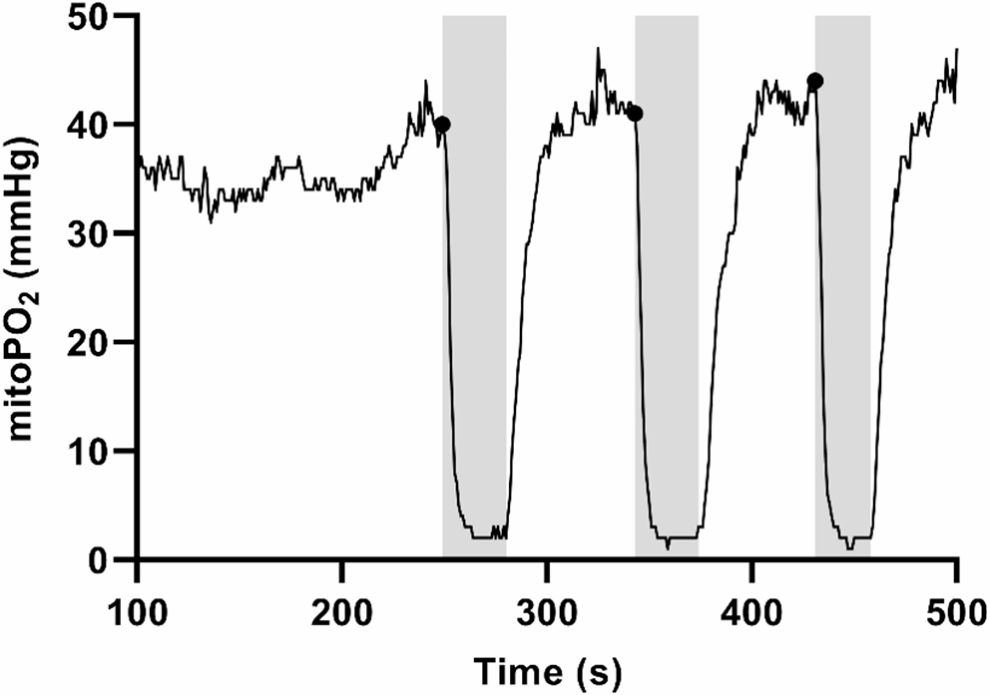
Overview of mitoPO_2_ data with mitoVO_2_ measurements. Start of mitoVO_2_ measurement (dots) and subsequent measurement (shaded gray) values are shown. All points outside the measurement are labeled ‘n/a’. Abbreviations: mitoPO_2_; mitochondrial oxygen tension

In this study mitoVO_2_ was measured using the COMET^®^ monitor (Photonics Healthcare BV, Utrecht, The Netherlands), which recorded mitoPO_2_ every second for 120 s. During each measurement, local pressure is applied to the COMET^®^ probe to create stop-flow conditions, causing a decline in mitoPO_2_. This decline is then used to calculate mitoVO_2_ (see Fig. 3 for representative data). A more detailed of how these data are used to determine mitoVO_2_ is provided later in this section.

### Datasets and labeling

Initially, the neural network-based software was developed and trained using a dataset from a previous clinical study of 39 subjects [13]. We later acquired a new dataset from an ongoing study at the Erasmus Medical Center in Rotterdam (registration number NL76685.078.21), which included 86 subjects. To organize this larger dataset we split it into two groups of 43 subjects each. One group (43 subjects) was combined with the original 39-subject dataset to create “Dataset A” (82 subjects total). The remaining group of 43 subjects was termed “Dataset B” and used solely for validating both the neural network and the Michaelis-Menten fit.

**Fig. 4:**
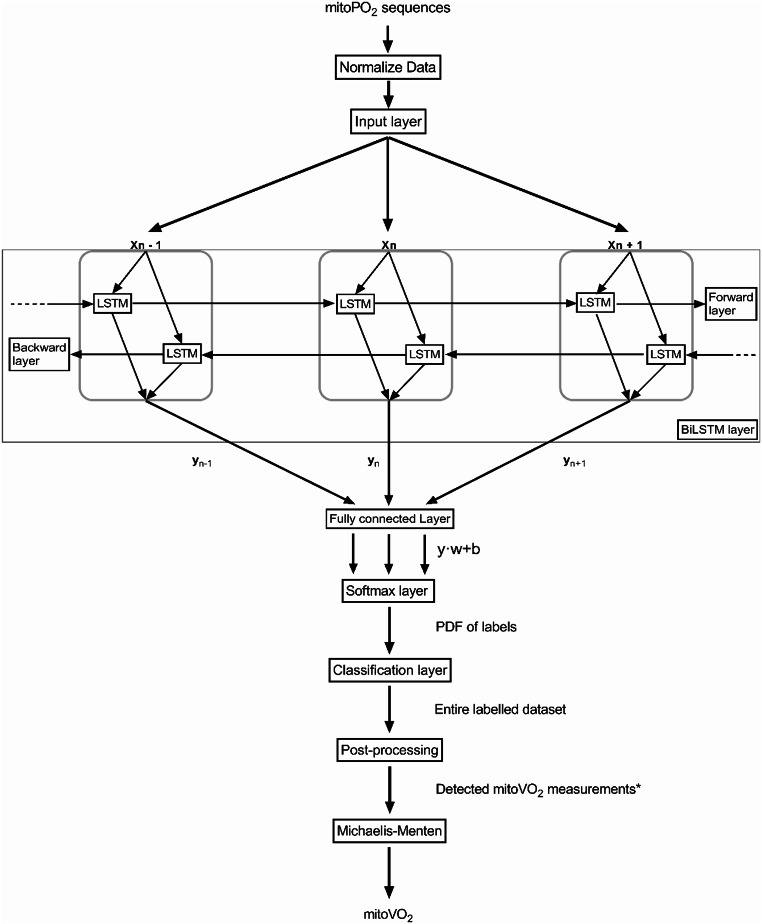
Schematic overview of network structure to automatically calculate the mitoVO_2_. Includes pre-processing steps, bilateral long short-term (LSTM) network, post-processing and Michaelis-Menten Fitting. *mitoVO_2_ measurements are detected as mitoPO_2_ values labeled ‘start’ with subsequent ‘measurement’ labeled mitoPO_2_ values. Abbreviations: b; bias, BiLSTM; bilateral long short-term network, LSTM; long short-term network, mitoPO_2_; mitochondrial oxygen tension, mitoVO_2;_ mitochondrial oxygen consumption, PDF; probability density function, w; weight

We used an in-house LabVIEW^®^ program to label each subject’s data, which contained multiple mitoVO_2_ measurements (see Fig. 4). Within each mitoVO_2_ measurement, all mitoPO_2_ data points were classified as ‘start’, ‘measurement’ or ‘n/a’. The label ‘start’ was assigned to the first clear drop in mitoPO_2_, and subsequent points were labeled ‘measurement’ until mitoPO_2_ rose again. All other points were labeled ‘n/a’. Details are in Supplementary Information 1.

Dataset A was labeled by two independent researchers, while Dataset B was labeled by three. Any discrepancies in Dataset A were resolved verbally. For Dataset B, we retained all three label sets to assess inter-rater variability, and used their consensus labels as the final reference for network validation.

### Neural network training and validation

The neural network was trained to perform mitoVO_2_ analyses (i.e., detecting ‘start’ and measurement’ points automatically), using only dataset A. To create a sufficient number of training examples, the measurements were subdivided into overlapping segments of 250 datapoints each, with a 240-point overlap (e.g., sample 1-250, sample 11–260, etc.) A 250-point window was chosen because it typically spans the entire descending slope of a given measurement, while a 240-point overlap ensures transitional data points are included in multiple segments, maximizing the effective training data. This sliding window approach artificially increases the sample size and accounts for transitions in mitoPO_2_ signals.

Bayesian optimization was employed to refine the network’s hyperparameters (Table 1). During each optimization iteration, the network was trained on a random 80% subset of Dataset A, with 10% held out for validation and 10% for internal testing. Classification error on the validation set served as the performance metric, and the optimizer was given a 30-hour time window to explore the parameter space. The hyperparameters fine-tuned during this process included the number of hidden units, initial learning rate, maximum epochs, and mini-batch size (Table 1). Importantly, Dataset B was not used at any stage of network training or hyperparameter tuning, thus serving as an external validation set.

**Table 1:** Hyperparameters optimized in training the network with given ranges

Hyperparameter	Range
No. Hidden units	50–80
Initial learn rate	0.001–1
Maximum epochs	2–4
Mini-batch size	60–100

Abbreviations: No.; number

### Network structure

The neural network was developed using MatLab^®^ 2022b (Deep Learning Toolbox version 14.5, The MathWorks Ltd, Natick, MA, United States). First, all data undergo z-score normalization, centering each variable to have a mean of zero and a standard deviation of one. These normalized data are then presented to a sequence input layer, with each data point represented as a vector x_n_ of length equal to mini-batch size.

Next, a bilateral long short-term memory (biLSTM) layer processes the input, using multiple long short-term memory (LSTM) blocks that exchange information both forward and backward. This design allows the network to learn long-term dependencies in both directions, effectively “memorizing” contextual information from prior and subsequent data points. The number of hidden units determines how many LSTM blocks are in each direction, thus controlling how much information is retained. The biLSTM’s output is a vector y_n_, where each data point has one value per hidden unit.

This output vector then enters a fully connected layer, where each element is multiplied by a learned weight and offset by a bias, resulting in a score for every possible label. These scores are then converted into probabilities through a softmax layer, and finally, a classification layer assigns one of three labels ‘start,’ ‘measurement,’ or ‘n/a’ to each data point.

### Post-processing labels

After the neural network assigns initial labels, a post-processing phase refines label accuracy by applying these rules.

1. If 1–2 consecutive data points are labeled ‘n/a’ but are surrounded by “measurement” labels, they are changed to “measurement.”
2. If 10 consecutive data points are labeled “measurement” without a preceding “start,” the first of those points is changed to “start.”
3. If two “start” labels occur within 30 data points, the second is discarded.
4. Any measurement that begins at a mitoPO_2_ < 20 mmHg or never reaches < 20 mmHg is excluded.
5. Any “n/a” data points between a “start” label and its subsequent “measurement” labels are relabeled as “measurement.”

These post-processing steps ensure that only a sustained drop in mitoPO_2_ qualifies as a valid “start,” preventing brief fluctuations from triggering extra “start” labels and eliminating very short or invalid measurements. Following these corrections, each “start” label and its consecutive “measurement” point form a single mitoVO_2_ measurement. A final quality control step ensures the Michaelis-Menten curve [17] is fit properly: we recenter the “start” label at the highest mitoPO_2_ value within the labeled “start” point and the next six points. If a sharp rise is detected in the subsequent three points, the “start” is shifted accordingly. Lastly, measurements showing a drop of more than 50 mmHg after the (re)defined start are considered invalid and removed. These adjustments yield a dataset that meets the criteria in Table 2, ensuring accurate Michaelis-Menten curve fitting.

**Table 2:** Requirements for mitoVO_2_ calculations on which post-processing is based

Part of measurement	Requirements
Start	1. 30 s apart 2. MitoPO_2_ > 20 mmHg 3. Highest MitoPO_2_ in 6 s range
Measurement	4. Duration > 10 s 5. No rise in mitoPO_2_ in first 3 s 6. Lowest mitoPO_2_ < 20 mmHg 7. > 50mmHg drop after first second

Abbreviations: mitoPO_2_; mitochondrial oxygen tension

### Michaelis-Menten fitting (mitoVO_2_ calculation)

Under stop-flow conditions (Fig. 1), mitoPO_2_ in the tissue drops due to oxygen consumption by both the tissue and the measurement itself. This decline establishes an oxygen gradient between the compressed tissue and surrounding tissue, driving diffusive influx. Consequently, the rate of PO₂ decrease under stop-flow can be modeled as:

1 $$\:dPO{}_{2}\left(t\right)/dt=\:-VO{}_{2}\left(t\right)-OCM\left(t\right)+DOI\left(t\right)$$

Where $\:dPO{}_{2}\left(t\right)/dt\:$is the rate of mitoPO_2_ decline, $\:VO{}_{2}\left(t\right)$ is the tissue’s oxygen consumption, $\:OCM\left(t\right)$ is the consumption measured and $\:DOI\left(t\right)$ represents diffusive oxygen influx [19]. Tissue oxygen consumption follows Michaelis-Menten kinetics, while DOI depends on an influx coefficient (Z) and a PO_2_ difference. Thereby providing the implemented equation:

2 $$\:dP{O}_{2}/dt\:=\:-\left({V}_{0}\:*\:P\:n\right)/({P}_{50}\:+\:{P}_{n})\:+\:Z({P}_{0}\:-\:{P}_{n})$$

As stated by Harms et al., V_0_ is the initial slope after tissue compression, P_n_ is the measured PO_2_ at time n, P_50_ is the PO_2_ at half V_max_, and P_0_ is the mean PO_2_ before compression [19]. Equation (2) is solved numerically (Runge-Kutta method in MatLab^®^ [23]), and the four unknown parameters (V_0_, P_50_, P_0_ and Z) are optimized using nonlinear least squares [23, 24]. A tangent is then drawn at the segment with the smallest derivative (steepest drop), yielding the maximum oxygen consumption (V_max_). V_max_ corresponds to mitoVO_2_ for that measurement. If multiple measurements are available for a subject, their mean V_max_ represents the subjects overall mitoVO_2_.

The complete software operates in two steps: first, the neural network identifies the start points of mitoVO_2_ measurements; second, the Michaelis-Menten approach described above calculates mitoVO_2_ from those labelled segments.

### Statistical analysis

After the neural network was trained and validated using Dataset A, Dataset B was used to assess how well the software-labeled data agreed with manual labels. To test repeatability, the software was run twice on Dataset B, and both outputs were compared.

We then evaluated two key metrics:

1. How closely the software’s “start” labels matched those identified by the raters (start point detection).
2. Whether the software’s calculated mitoVO_2_ values aligned with the rater-derived values, using Bland-Altman analysis [25] (mitoVO_2_ comparison).

#### Start point detection

Start points were compared among three raters, their manual consensus and the neural network outputs (including the second software run). Start points were considered identical if they fell within five samples of each other (e.g. Rater 1 labels sample 100, and Rater 2 labels sample 105). A histogram of these offsets (-5 to + 5) was created, and the absolute and relative rates of matching start points were calculated. We also checked that the network’s agreement with the manual consensus did not deviate by more than five points from the raters’ mutual agreement. The accuracy was then calculated by dividing the amount of points within a certain offset by the total amount of start points. Four levels of accuracy were calculated based on the offset: zero (acc0), 1 sample (acc1), 3 samples (acc3) and 5 samples (acc5).

#### mitoVO_2_ comparison

For each subject in Dataset B, the mean mitoVO_2_ was calculated by each rater, by the manual consensus, and by the software. A Bland-Altman analysis [25] was then performed to evaluate agreement, recognizing there is no single “gold standard” for comparison. Mean bias representing any systematic difference was calculated, and its 95% confidence interval (CI) was obtained via a paired sample t-test, assuming normality under the central limit theorem [26]. The limits of agreement (LoA) define the interval in which 95% of the method differences lie. Extended LoA calculations were used to handle repeated measurements with potentially different replicate counts for each rater [27].

To determine whether the software showed comparable mitoVO_2_ values to the manual consensus, a predefined comparability range was established, derived from the commonly used non-inferiority margin. In this study, raters or methods could not be deemed superior or inferior to each other because of the absence of a gold standard. For comparability, the 95% CI of bias between the software and manual consensus must lie within the predefined range. The comparability range for the software and manual consensus was set at +/- 0.3 mmHg s^− 1^. Identical ranges were also established for the LoA compared to the inter-rater LoA.

## Results

### Data description

Dataset A consisted of 805 mitoVO_2_ measurements from 82 subjects. Dataset B included 43 subjects, yielding a total of 327 mitoVO_2_ measurements. Within Dataset B, two subjects had no valid mitoVO_2_ measurements and were excluded from further analysis.

### Network hyperparameters

Bayesian optimization of the network was conducted for 30 h, resulting in an overall performance of 94.2% on the test set (the 10% portion of Dataset A not used for training or validation). The final hyperparameters values are listed in Table 3.

**Table 3:** Final hyperparameter values after bayesian optimization on dataset A

Hyperparameter	Range
No. Hidden units	55
Initial learn rate	0.0137
Maximum epochs	3
Mini-batch size	80

Abbreviations: No.; number

### Start point detection

**Fig. 5:**
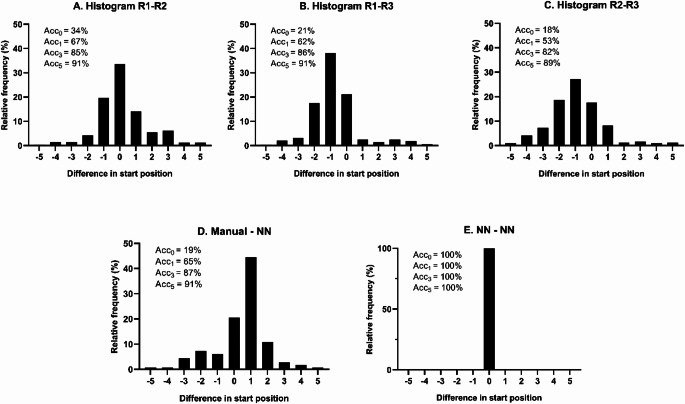
mitoVO_2_ analysis: difference in start point detection between raters (**A-C**), neural network – manual consensus (**D**) and NN-NN (**E**). Abbreviations: Acc_0_; Offset of 0 sample (exact matches), Acc_1_; Offset of ± 1 sample, Acc_3_; Offset of ± 3 samples, Acc_5_; Offset of ± 5 samples, NN; neural network, R; rater

Rater 1, Rater 2, and Rater 3 respectively identified 325, 309, and 317 total start points for mitoVO_2_ measurements. When examining the overlap of these start points within a ± 5-sample range, 290 (89%) of Rater 1’s points aligned with Rater 2’s, 297 (91%) with Rater 3’s, and 282 (89%) of Rater 2’s aligned with Rater 3’s. Figure 5A–C depicts the distributions of these differences among the three raters and corresponding accuracies.

Consensus labeling by all three raters yielded 327 start points in Dataset B. Of these, 297 (91%) were matched within a ± 5-sample range by the neural network–based software, following post-processing. In addition, the neural network identified 19 start points that were not marked by any rater. Figure 5D shows the distribution of differences between the manual consensus labels and the neural network labels, along with corresponding accuracies.

When the software was run twice on the same data, it produced identical start points, resulting in a zero-sample difference accuracy of 100% (acc₀), as shown in Fig. 5E.

### mitoVO_2_ values

**Fig. 6:**
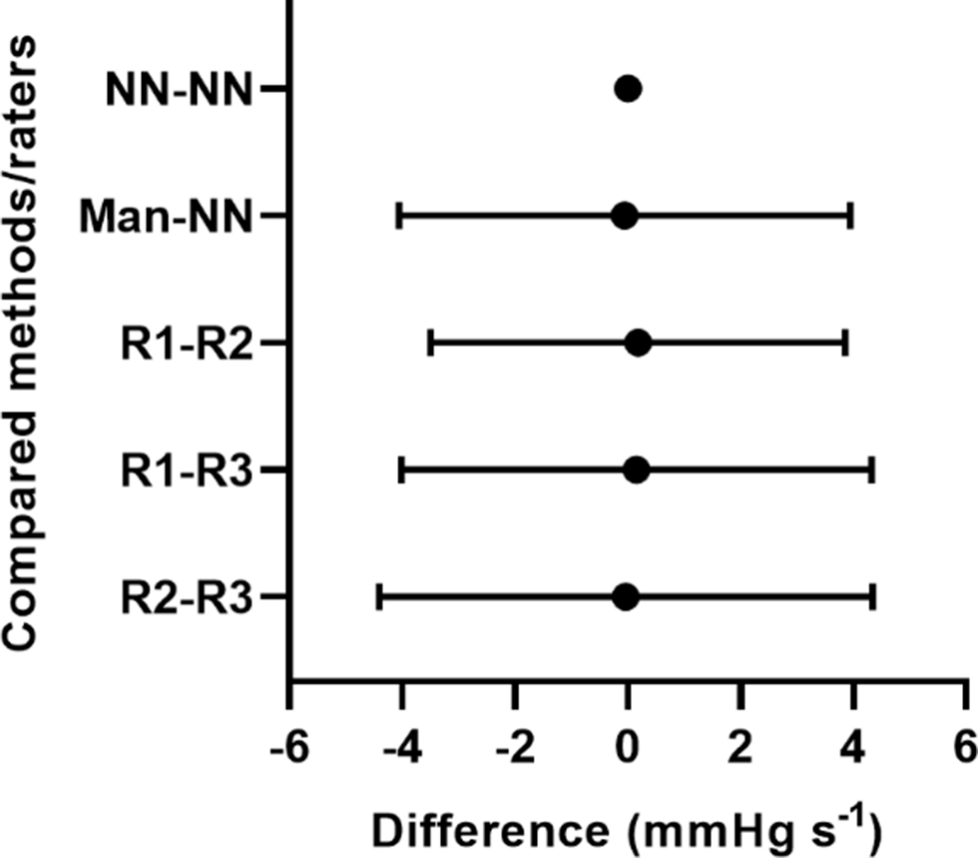
mitoVO_2_ bias and limits of agreement between different methods and raters. NN: Neural network- based software. Man: Manual consensus. R1: Rater (1) R2: Rater (2) R3: Rater Abbreviations: Man; manual consensus, NN; neural network, R; rater

Table 4 summarizes the mean mitoVO_2_ values (± SD) obtained by each individual rater, the manual consensus, and the neural network–based software. The bias between the software and the manual consensus was − 0.057 mmHg s^− 1^ (95% CI -0.15–0.27 mmHg s^− 1^), with LoA from − 4.06 to 3.95 mmHg s^− 1^. Re-running the software produced identical results (Fig. 6).

**Table 4:** Mean mitoVO_2_ values as determined by the raters, manual consensus and the neural network-based software

Rater/method	mitoVO_2_ (mmHg s^− 1^) [mean SD]
Rater 1	6.61 ± 2.35
Rater 2	6.43 ± 2.51
Rater 3	6.45 ± 2.48
Manual consensus	6.56 ± 2.47
Neural network-based software	6.63 ± 2.42

Abbreviations: mitoVO_2_; mitochondrial oxygen consumption

Pairwise biases among the three raters (Rater 1–2, Rater 1–3, and Rater 2–3) were 0.18, 0.13, and − 0.031 mmHg s^− 1^, respectively. The corresponding LoAs adjusted for repeated measurements, ranged from − 3.50 to 3.85, -4.02 to 4.32 and − 4.40 to 4.34 mmHg s^− 1^ (Fig. 6). The complete Bland-Altman plots are provided in Appendix 1.

## Discussion

The software developed in this study can automatically analyze mitoVO_2_ values from the COMET^®^ output and shows strong performance. Its effectiveness is reflected in the neural network’s identification of 91% of mitoVO_2_ start points within five samples of the manual consensus a rate equivalent to the highest agreement between the two individual raters. Moreover, each run of the neural network consistently produced identical start points, thereby removing inter-user variability and ensuring reproducibility. The resulting bias between the software’s outputs and the manual consensus was, on average, smaller than the bias between individual raters, with overlapping LoA. Multiple runs of the software consistently yielded the same mitoVO_2_ values, illustrating its potential to improve the consistency of analyses across research groups. By employing this software, researchers can obtain more comparable results, avoiding the variability we observed when different observers interpreted start points in the same dataset.

After the neural network consistently identified the start point, we used a modified Michaelis-Menten fit to calculate mitoVO_2_. While the standard Michaelis-Menten equation assumes an in vitro, closed-respirometry scenario and thus overlooks oxygen diffusion back into the measurement volume, the modified approach by Harms et al. [17, 19] accounts for both oxygen consumption and tissue diffusion in vivo. Other computational strategies, such as the sigmoid-based method proposed by Baumbach et al. [18], could also yield accurate results in certain settings. However, we favor the modified Michaelis-Menten equation because it closely reflects underlying biological processes and explicitly incorporates oxygen diffusion from surrounding tissue [17, 19].

By embedding our neural network’s output into a physiologically grounded model, we provide a method that researchers can adopt to analyze mitoVO_2_ data more consistently. This is especially valuable when clinical measurements occur outside tightly controlled laboratory environments. Nevertheless, we acknowledge that no single model can capture all physiological nuances. Future iterations potentially informed by larger datasets and multi-center collaboration may refine or expand on the present approach.

## Future directions

Although we observed no major discrepancies between user consensus and our software’s outputs, we emphasize that this is only one approach among potential methods for analyzing mitoVO_2_. By making the neural network-based application freely available (see Supplementary Material S2 for the software and Supplementary Material S3 for installation instructions), we aim to promote consistency and comparability of mitoVO_2_ data across research groups. Encouraging widespread use of this software could reduce observer-related variability, as demonstrated by the differences we noted within our own group. This, in turn, should yield greater uniformity in clinical and research findings, ultimately enhancing the value and clarity of future mitoVO_2_ studies.

Moreover, the COMET^®^ monitor also permits measuring mitochondrial oxygen delivery (mitoDO_2_), as introduced by Baumbach et al. [18]. We opted not to include mitoDO_2_ in the current iteration due to its additional complexity and limited availability of mitoDO_2_ data in our center. In subsequent versions, we plan to incorporate mitoDO_2_ analyses, expanding the software’s utility for assessing diverse aspects of mitochondrial function. Our strategy is to integrate feedback and data from multiple research centers, steadily refining the network’s accuracy and feature set.

## Limitations

A notable limitation of the software is its periodic need for user intervention to identify major abnormalities. Occasionally, the neural network may mislabel a mitoVO_2_ measurement, potentially skewing results. However, these errors are generally easy to spot using the software’s visualization tools, which display each mitoVO_2_ curve identified by the network.

The limitations described here can be viewed as initial hurdles that we expect to address in future iterations of the neural network. Nonetheless, we considered it important to publish and freely provide this neural network-based software so that researchers can begin adopting a more standardized approach for analyzing mitoVO_2_. Future updates built on larger datasets and inter-center collaborations will refine the software further, expanding both its robustness and its alignment with clinical and research needs.

## Conclusion

In conclusion, this study successfully developed a neural network–based software that automatically analyzes mitoVO_2_ data, offering high consistency and removing inter-user variability. By proposing a method of standardized mitoVO_2_ analysis across different research groups, the software enhances result comparability, which is especially critical given the variability observed even within a single team. To encourage widespread use, we provide the application free of charge, streamlining both usability and comparability of mitoVO_2_ measurements.

## Appendix

**Figure:**
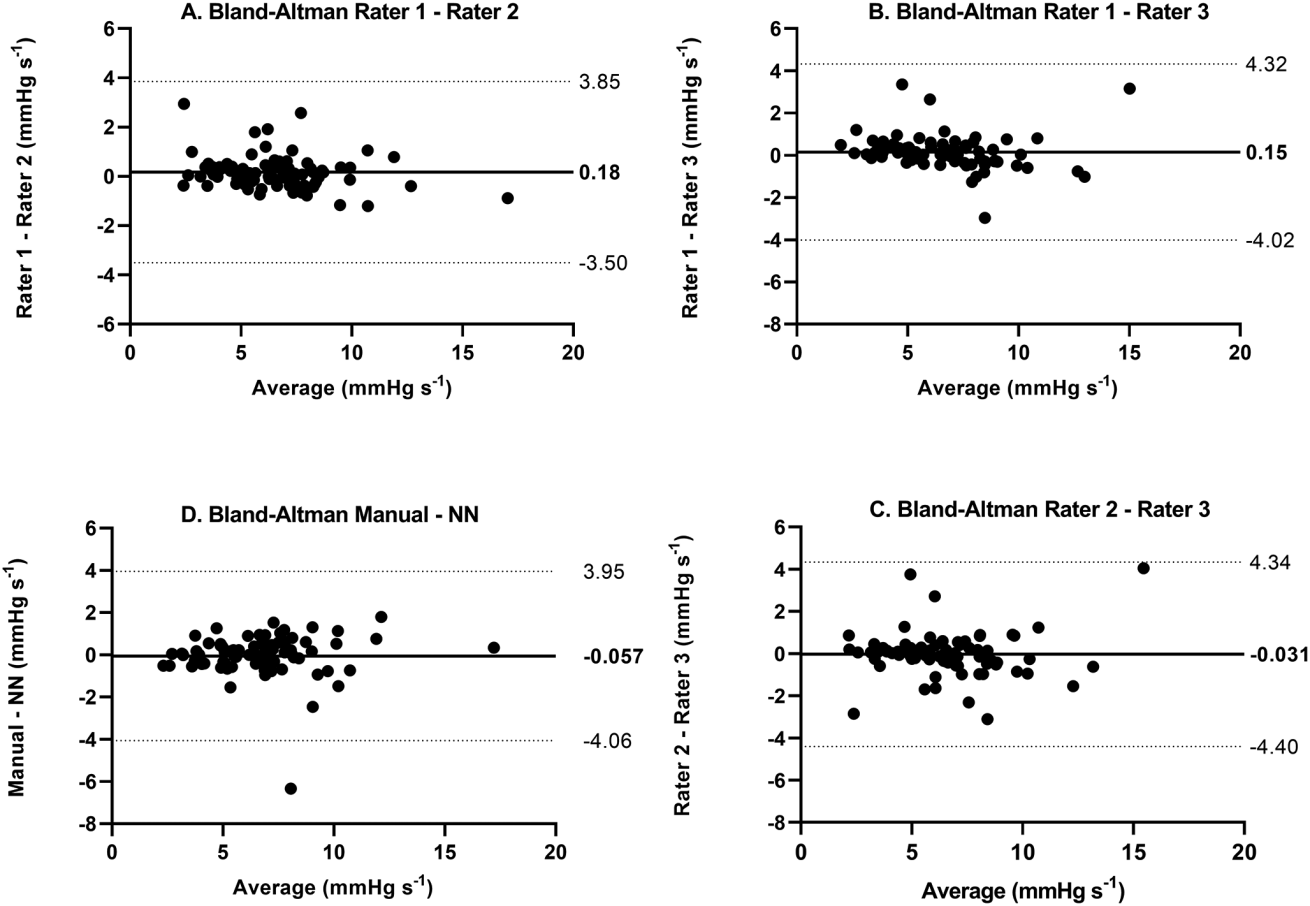
Fig. A1 Bland-Altman plot comparing different raters (**A**-**C**) and the manual consensus to the neural network (**D**); MitoVO_2_

## Electronic supplementary material

Below is the link to the electronic supplementary material.

Supplementary Material 1

Supplementary Material 2

Supplementary Material 3

## Data Availability

The datasets generated and/or analyzed during the current study contain personal patient data and are subject to the General Data Protection Regulation (GDPR) in Europe. Therefore, they are not publicly available. Data can be made available from the corresponding author upon reasonable request and with adherence to GDPR guidelines.
