# Supplementary material for: Automated mitochondrial oxygen consumption (mitoVO2) analysis via a bi-directional long short-term memory neural network
Source: J Clin Monit Comput. 2025 Mar 30;39(5):947–56. doi: 10.1007/s10877-025-01291-1 (PMC12474646; doi:10.1007/s10877-025-01291-1)
Supplement: Supplementary file 1 — Supplementary Material 1 [file 10877_2025_1291_MOESM1_ESM.pdf]

Article title: Automated Mitochondrial Oxygen Consumption (mitoVO_2) Analysis via a Bi-Directional Long Short-Term Memory Neural Network

Journal name: Journal of Clinical Monitoring and Computing

Author names: C.J. de Wijs[1*], J.R. Behr[1,2], L.W.J.M. Streng[1], M.E. van der Graaf[1,2], F.A. Harms[1], E.G. Mik[1]

Affiliations:

[1] Department of Anesthesiology, Erasmus Medical Center, Rotterdam, the Netherlands

[2] Faculty of Mechanical Engineering, Delft University of Technology, Delft, the Netherlands

Corresponding author: C.J. de Wijs, e-mail: c.dewijs@erasmusmc.nl

PROTOCOL: MITOVO$_2$ ANALYSES THROUGH LABVIEW

Installing LabVIEW

1. Download and install LabVIEW 2013 32bit runtime version. Note this software only works on windows computers. For quick access follow the hyperlink: https://www.ni.com/nl-nl/support/downloads/software-products/download.labview.html#359538

Opening the program

2. On both the COMET USBs and the COMET laptops there is a file named *MM fitter RUv2,* open this file and open the program within also labelled *MM fitter RUv2 highlighted in blue as i*llustrated in figure 1.

Fig. 1; image of MM fitter RU v2 file

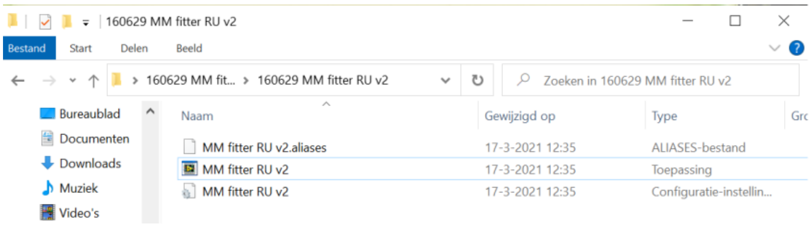

3. The program will then prompt you to select the COMET export file you wish to open. Open this file.
4. The program will then load the COMET data that you have selected. The window will show a chart with the comet data. As illustrated below in figure 2. NOTE FILE OPEN FROM WITHIN THE SYSTEM DOES NOT WORK. Thus if you want to do a new data set you must restart the program.

Fig.2: chart as first shown by the LabVIEW program.

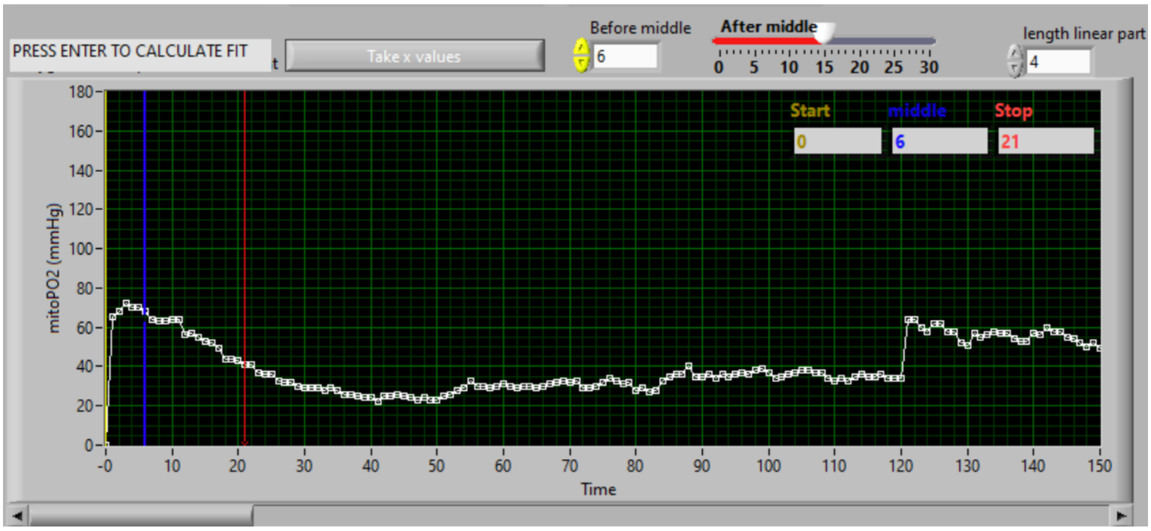

Measuring the mitoVO₂

1. The lines can be found in the farthest left corner of the program. You have to shift the bar to the left to reach these points.
2. In order to measure the mitoVO₂ the blue middle line must be dragged and placed on the inflection point of the mitoVO₂ curve as shown below.
3. The red stop line must be placed on the stationary phase of the curve where the mitoPO₂ is less than 10. To do this you must utilize the shifter labeled '*after middle*'. This is portrayed in figure 3.
4. The yellow line does not need to be shifted.

Fig. 3: Illustrates the positioning of the middle and stop lines.

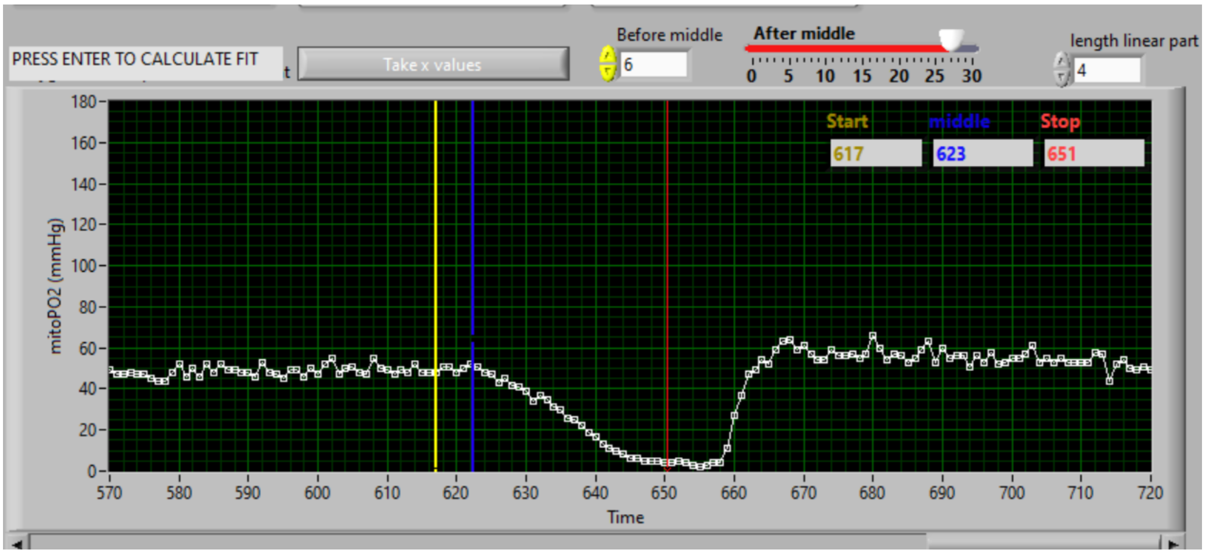

5. Once you have selected the middle and end point press enter. The program will then calculate two Michaelis-menten (MM) curves, the yellow curve represents the *"MM fit VO₂ max and Y0 fixed"*, the red curve represents *"First MM minimum"* and the green represents the slope. Shown in figure 4

Fig. 4 illustrates the MM curves that the program calculates.

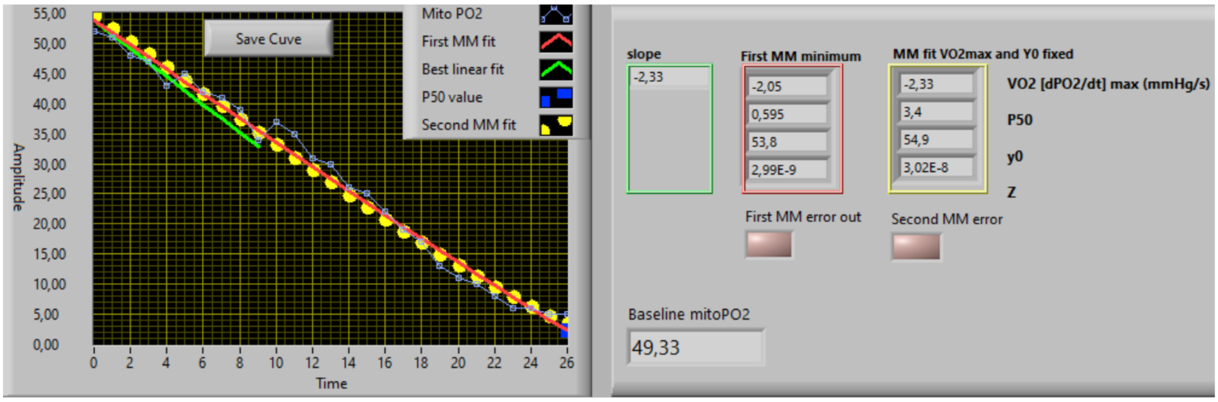

6. When examining the output of the program it is vital to evaluate if the yellow MM fit does not deviate too much from the mitoPO_2 points as illustrated in figure 4.
7. If it, you are unsatisfied with the curve output you can go back to step 3 and adjust the middle and end point. However, it is vital that the middle point (indicated by the blue line) is on the inflection point of the mitoVO_2 curve (preferably the highest value should be chosen). Then again press enter.
8. Once satisfied with the curve press save curve, and it will output the data in a legible format, please save this data in the same place that your COMET data is stored.
9. If the Save function of the LabVIEW system does not work, press *windows + shift +s* for the snipping tool and save an image similar to figure 4 so that you can always reference your original output.
10. Lastly, the mitoVO_2 that is utilized in data analysis is the *“MM fit* VO_2 *max and Y0 fixed”* this also corresponds to the slope. (For figure 4 the mitoVO_2 is -2.33mmHg/s)
