# Supplementary material for: Automated mitochondrial oxygen consumption (mitoVO2) analysis via a bi-directional long short-term memory neural network
Source: J Clin Monit Comput. 2025 Mar 30;39(5):947–56. doi: 10.1007/s10877-025-01291-1 (PMC12474646; doi:10.1007/s10877-025-01291-1)
Supplement: Supplementary file 3 — Supplementary Material 3 [file 10877_2025_1291_MOESM3_ESM.pdf]

Erasmus MC

Supporting Information S3: Installation manual

Article title: Automated Mitochondrial Oxygen Consumption (mitoVO_2) Analysis via a Bi-Directional Long Short-Term Memory Neural Network

Journal name: Journal of Clinical Monitoring and Computing

Author names: C.J. de Wijs[1*], J.R. Behr[1,2], L.W.J.M. Streng[1], M.E. van der Graaf[1,2], F.A. Harms[1], E.G. Mik[1]

Affiliations:

[1] Department of Anesthesiology, Erasmus Medical Center, Rotterdam, the Netherlands

[2] Faculty of Mechanical Engineering, Delft University of Technology, Delft, the Netherlands

Corresponding author: C.J. de Wijs, e-mail: c.dewijs@erasmusmc.nl

The application in which the automated MitoVO$_2$ software is embedded can be downloaded from through the supporting information. In this manual, the steps to download the software are explained.

1. Make sure your device is connected to the internet.
2. Click on **the file link provided as supporting information S4** to download the application.
3. Double click on "MyAppInstaller_web.exe".
4. If your computer asks for permission. Click "Yes".
5. The following screen should pop-up:

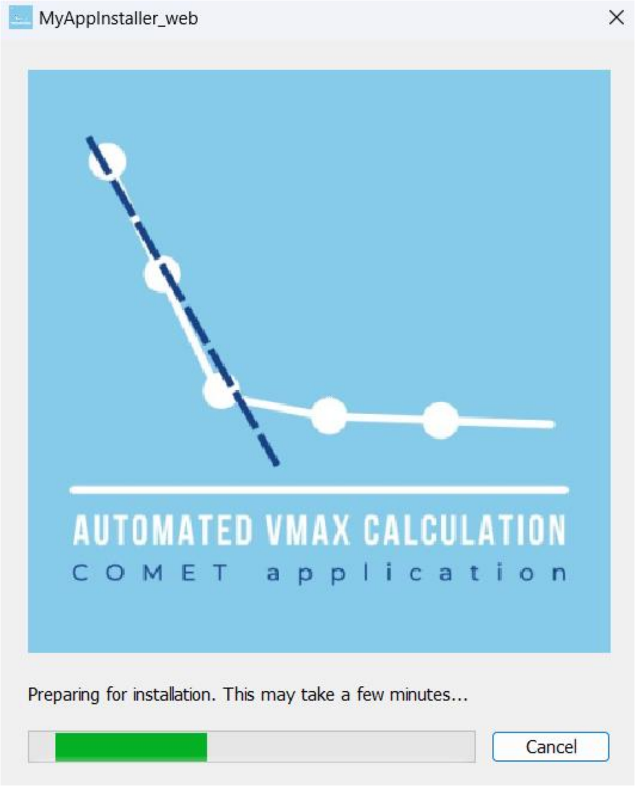

Figure 1 Download screen

6. On the following screen, click "Next".
7. Select a destination folder. The application will be saved here. If you are not sure where to save the application, use the default location by clicking "Next". If the folder you chose is not valid, click on "restore default" and click "Next".

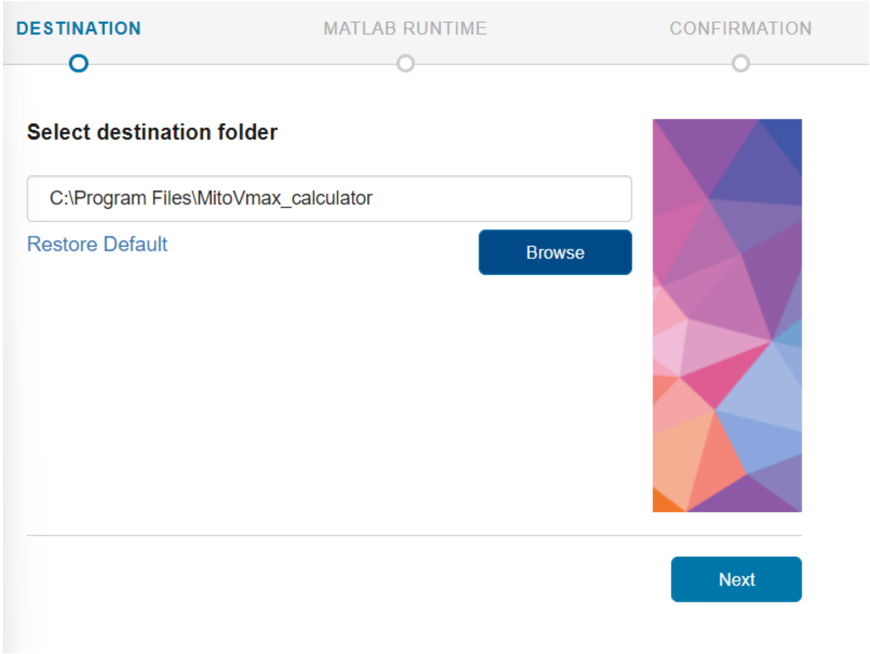

Figure 2 Program saving screen

8. To install the application, MatLab Runtime is installed. Select a destination folder. If you are not sure where to save the application, use the default location by clicking “Next”. If the folder you chose is not valid, click on “restore default” and click “Next”.

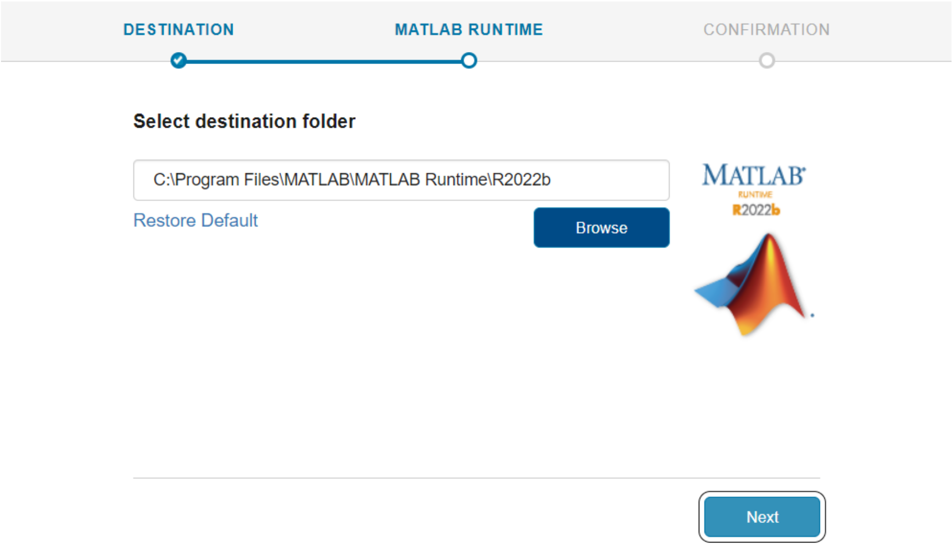

Figure 3 Runtime destination folder

9. Read the terms of the license agreement. Then accept by clicking the circle before “Yes” and click “Next” to continue.

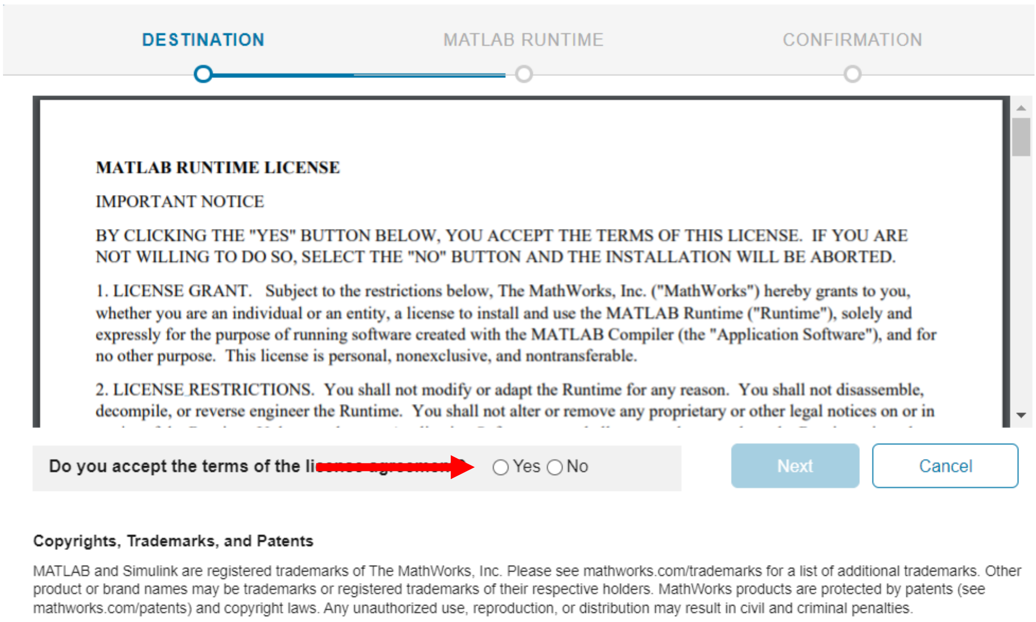

Figure 4 Terms of agreement

10. Check if the destination folders are correct. If so, click “Begin install”.

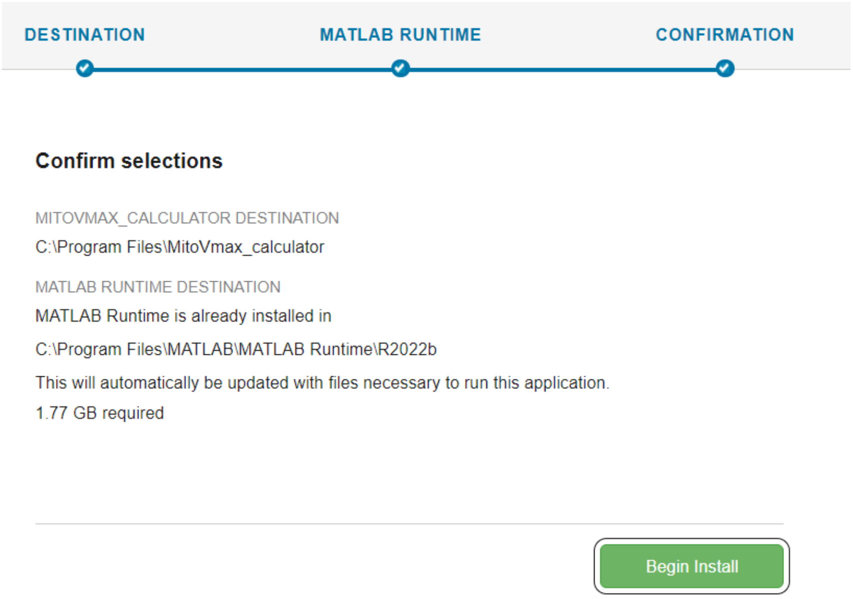

Figure 5 Confirmation screen

11. The application will now be installed on your device. This might take some time.

12. To open the application, search “AutomatedVO2Calculator” in your device’s search bar or go to the destination you specified and click “AutomatedVO2Calculator.exe”
